# Influence of Removing or Leaving the Prosthesis after Regenerative Surgery in Peri-Implant Defects: Retrospective Study: 32 Clinical Cases with 2 to 8 Years of Follow-Up

**DOI:** 10.3390/ijerph18020645

**Published:** 2021-01-13

**Authors:** Víctor Astolfi, Alberto Gómez-Menchero, José Vicente Ríos-Santos, Pedro Bullón, Francisco Galeote, Blanca Ríos-Carrasco, Beatriz Bullón de la Fuente, Mariano Herrero-Climent

**Affiliations:** 1 Department of Periodontics and Dental Implants, Universidad de Sevilla, 41009 Seville, Spain; doctorvastolfi@gmail.com (V.A.); alberto@gomezmenchero.com (A.G.-M.); pbullon@us.es (P.B.); pacogaleote.com@gmail.com (F.G.); brios@us.es (B.R.-C.); beatrizbullon@hotmail.com (B.B.d.l.F.); 2 Porto Dental Institute, 4150-518 Porto, Portugal; dr.herrero@herrerocliment.com

**Keywords:** peri-implantitis, regenerative surgery, peri-implant defects

## Abstract

Purpose: The aim of this retrospective study was to compare the influence of removing or not removing a prosthesis after regenerative surgery on peri-implant defects. Methods: Two different groups were compared (Group 1: removing the prosthesis; Group 2: maintaining the prosthesis), analyzing radiographic bone filling (n = 32 implants) after regenerative treatment in periapical radiographs. The peri-implant defects were measured before and after regenerative treatment using Bio-Oss^®^ (Geistlich Pharma, Wohhusen, Switzerland) and a reabsorbable collagen membrane (Jason^®^, Botis, Berlin, Germany), the healing period was two years after peri-implant regenerative surgery. Statistical analysis was performed, and a Chi square test was carried out. To determine the groups that made the difference, corrected standardized Haberman residuals were used, and previously a normality test had been applied; therefore, an ANOVA or Mann–Whitney U test was used for the crossover with the non-normal variables in Group 1 and Group 2. Results: The results obtained suggest that a regenerative procedure with xenograft, resorbable membrane, and detoxifying the implant surface with hydrogen peroxide form a reliable technique to achieve medium-term results, obtaining an average bone gain at a radiographic level of 2.84 mm (±1.78 mm) in patients whose prosthesis was not removed after peri-implant bone regenerative therapy and 2.18 mm (±1.41 mm) in patients whose prosthesis was removed during the healing period. Conclusions: There are no statistically significant differences in the response to treatment when removing or keeping the prosthesis after regenerative surgery in peri-implant defects.

## 1. Introduction

Currently, dental implants present very high success rates, and results tend to be remain successful for years [1,2]. Most longitudinal studies have reported survival rates of around 90–95% for periods of 5–10 years. However, this treatment is not free of biological complications (mucositis and peri-implantitis) or adverse results. The type of inflammatory lesions that may develop in the tissues around the implants are known as peri-implant diseases. While mucositis is defined as an inflammation of the peri-implant mucosa with the absence of marginal bone loss, peri-implantitis is a pathological condition that occurs in the peri-implant tissues, which is characterized by the inflammation of the peri-implant mucosa and progressive loss of supporting bone [3].

Epidemiological data refer to a prevalence of peri-implant pathology, around 19–65% [4], which may endanger the longevity of dental implant restorations. Studies such as the one carried out by Rodrigo et al. [5] in 2018 show a 51% prevalence of peri-implant diseases in the Spanish population, suggesting that one out of two implants placed will develop peri-implant pathologies. Risk factors that trigger these diseases should be taken into account, such as a previous history of periodontitis and inadequate maintenance and oral hygiene, which are indicators of peri-implantitis. However, there are other factors with limited evidence in the literature, such as smoking, diabetes, the presence of at least 2 mm of keratinized mucosa, prosthetic characteristics, or genetic factors (genetic polymorphisms interleukin 1 (IL-1) [6].

The objective of successful treatment of peri-implant diseases should be the resolution of the inflammation and the preservation of the supporting bone, and in cases where bone loss has been obtained, recovery of the lost bone, by regenerative treatment [7]. Several therapeutic modalities have been classically suggested for the treatment of peri-implant diseases. Non-surgical treatment, which consists of mechanical debridement, ultrasonic or laser devices, alone or combined with antiseptic and/or antibiotic agents, has not demonstrated the resolution of the disease due to inconsistent results. Surgical therapy, in addition to the debridement of peri-implant tissues, consists of applying resective or regenerative surgical techniques that have shown greater effectiveness in terms of disease resolution as demonstrated by preclinical and clinical studies [8,9,10].

The decision as to whether surgical therapy should be regenerative or resective is conditioned by the configuration of the peri-implant bone defect. A bone defect is defined as the loss of marginal bone around the implant; these defects are classified as intraosseous or supraosseous according to the pattern of bone resorption. When the residual bone is uneven or there are supraosseous defects, resective surgery [11], with or without implantoplasty, has had satisfactory results. Resective surgical therapy is the best choice to treat horizontal bone loss and moderate bone defects (less than 3 mm) in order to reduce the depth of the peri-implant pocket and guarantee better oral hygiene. However, in cases where the morphology of the defect is favorable (three or four wall bone defects), regenerative techniques can be justified. With this therapy, the concept of guided bone regeneration (GBR) is achieved by placing a barrier membrane and bone filling through substitutes (Dahlin et al. 1988) [12]. The objective of this therapeutic option is the restoration and maintenance the peri-implant tissues, as well as improving the chances of obtaining reosteintegration. To help clinicians select the appropriate surgical modality, various classifications of peri-implant defect types have been proposed. Authors such as Schwarz et al. [13] and Serino and Strom [14] state that most of the defects are circumferential; however, a recent study by Monje et al. [15] discovered through previous radiographic studies that more than 50% of peri-implantary defects were two or three walls with no vestibular table.

However, one of the most important factors in the long-term success of oral implants is an adequate maintenance of the peri-implant tissues. A causal relationship has been demonstrated between the accumulation of bacterial plaque and the development of inflammatory changes in the soft tissues around oral implants [16]. If this condition is not treated, it can lead to the progressive destruction of the tissues supporting the implant (peri-implantitis) and finally to the implant’s failure [17].

Maintenance is a key success factor in the treatment of peri-implant infections, and patients who are susceptible to developing infections should be regularly examined and receive appropriate supportive therapy. Infection evolves slowly and gradually, and it is crucial that it can be intercepted early to prevent damage to peri-implant tissues [18].

The main objective of this study is to determine the influence on the healing process of leaving or not leaving the prosthesis after regenerative surgical treatment of bone defects produced by peri-implant pathology [19,20,21].

## 2. Materials and Methods

This study comprised a series of cases in which regenerative therapy was performed for the treatment of bone defects caused by previous peri-implant pathology, in accordance with the Declaration of Helsinki, its Tokyo and Venice amendments, the Spanish Royal Decree of 16 April 1993, and the Good Clinical Practice in force in the European Union (BCP of July 1990). The protocol was approved by the Ethical Committee Nº:112018 of the Andalusian Regional Health Council, signed in Seville on 14 December 2018. All patients included in the study were informed about the purpose of the research and were required to sign an informed consent.

### 2.1. Study Sample

This is a retrospective study, which was carried out at the Department of Periodontology and Implants of the University of Seville. Radiographic bone filling was evaluated in 28 patients with peri-implant disease affecting 32 implants, who presented infrabony defects susceptible to regenerative surgical therapy. Two study groups were determined:

A: Maintenance of the prosthesis after peri-implant bone regenerative surgery.

B: Removal of the prosthesis after peri-implant bone regenerative surgery with a minimum of 8 weeks for healing to facilitate surgical access for the operator.

The following inclusion criteria were applied: patients with peri-implantitis susceptible to regenerative therapy, which was defined according to the criteria of Renvert et al. as the presence of profuse bleeding and/or suppuration on soft probing (0.15 Ncm), a probing depth ≥6 mm, and evidence of radiographic bone loss ≥3 mm measured with reference to the initial radiograph, from the most coronal portion of the defect to the most infrabony part of the implant based on periapical radiograph [6]. In cases where access was complex, the examiner removed the prosthesis for greater diagnostic precision.

Aged between 18 and 80 years without the presence of infectious diseases at the time of implant placement or during the maintenance phase, with a minimum follow-up period of 2 years, all patients who presented implants with mobility or peri-implant bone defects ≤3 mm were excluded, as were all those who did not sign the informed consent.

### 2.2. Clinical Assessment

The following clinical parameters and indices were recorded at the start of the study (T0) (day of diagnosis of the peri-implant pathology) and in the postoperative period at 24–96 months (T1), in six peri-implant locations by a single examiner (AG-M).

- Probing depth (PD) (in mm): with a North Carolina periodontal probe applying a pressure of 0.15 Ncm.
- Modified Sulcular Bleeding Index (mBI): registered from 0 to 3, according to the criteria of Mombelli et al. and the extension and severity of bleeding on probing (BOP) [22].
- Modified plaque index (mPI): registered according to the O’Leary index that represents the average percentage of dental surfaces affected by bacterial plaque [23].
- Mucosal recession (REC): defined as the distance (in mm) from the implant platform as a stable mark and margin of the mucosa.
- Suppuration (SUP): around the implants according to the dichotomous scale (0/1), using the University of North Carolina probe 15, applying a force of 0.15 Ncm.

General Data

- Smoking habits: No, <10/day, Yes, >10/day.
- Systemic diseases: arterial hypertension, diabetes, osteoporosis, cardiovascular disease, HIV, respiratory infections.
- Periodontal disease: No, Mild (Clinical Attachment Level—CAL 1–2 mm), Moderate (CAL 3–4 mm), Advanced (>5 mm) [23].
- Maintenance: No, Every 4 months, Every 6 months, Every 12 months.

Implants Date

**Figure 1:**
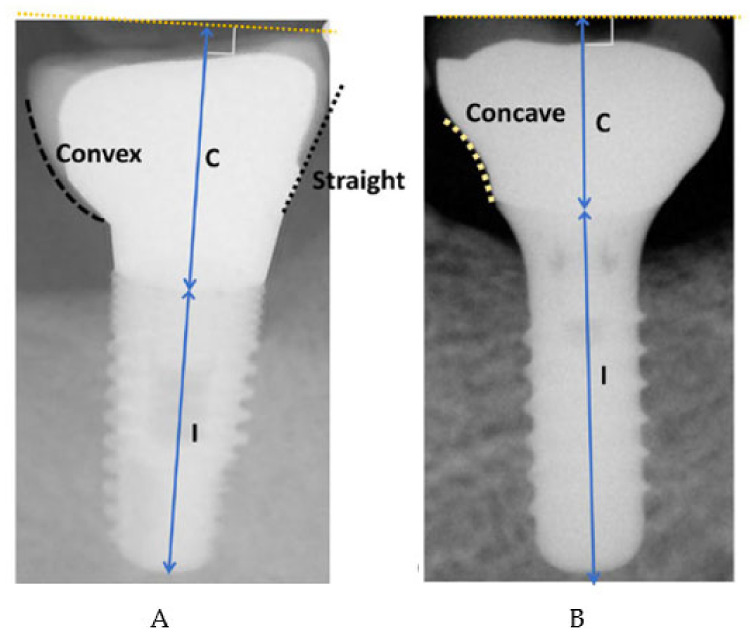
(**A**) Incorrect passive fit. (**B**) Correct passive fit. Images taken from Yi et al. [26]. C: crown. I: Implant.

- Date of implant placement.
- Surface: 1: minimally rough (0.5–1 µm), 2: moderately rough (1–2 µm), 3: rough (> 2 µm), based on the Albrektsson and Wenneberg’s 2004 classification [24].
- Location: Following World Dental Federation (FDI) nomenclature.
- Type of prosthesis: screwed/cemented single crown, screwed/cemented fixed partial prosthesis, hybrid prosthesis, metal–ceramic fixed prosthesis, overdenture.
- Diameter and length of each implant.
- Type of connection: 1: bone level, 2: tissue level, 3: external connection.
- Date of prosthesis placement: 1: immediate loading (24 h–1 week), 2: early (1–8 weeks), 3: conventional (>2 months), according to the criteria of Esposito et al. [25].
- Overcontoured or non-overcontoured prostheses: assessed by periapical radiographs and clinical evaluation by a calibrated examiner, where an overcontoured prosthesis is more associated with inflammation and the retention of bacterial plaque compared to prostheses with an adequate contour (Figure 1).

**Figure 2:**
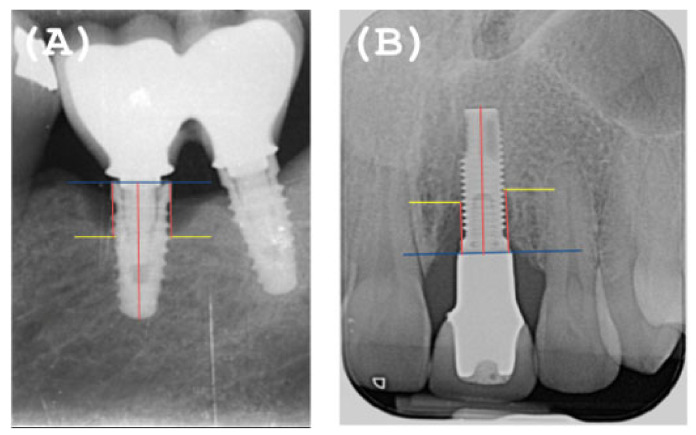
(**A**) Incorrect passive fit. (**B**) Correct passive fit.

Good passive fit or not: assessed by periapical radiographs taken with a parallelizer, where the absence of a “gap” between the prosthetic restoration and the implant was evaluated (Figure 2).

### 2.3. Satisfaction Questionnaire

After the control visit (T1) on 8 July 2019, patients were asked to subjectively evaluate the mastication, phonetics, and aesthetics of the implant-supported restoration and oral hygiene through the following question: “How do you evaluate your implant-supported restoration in terms of mastication, phonetics, aesthetics, and possibility of cleaning, good, acceptable, or bad?”

### 2.4. Definition of Disease Resolution

The success of the treatment was determined when, after 24 months of regenerative therapy, the following criteria were met:

- Absence of BOP after soft probing (0.15–0.2 Ncm).
- Probing Depth ≤5 mm.
- Progressive radiographic bone gain (±0.5 mm).

### 2.5. Radiographic Evaluation

Peri-implant radiographic bone loss (MBL) was determined at T0 (day of diagnosis of peri-implant pathology) and T1 (day of review after at least two years of peri-implant surgery) by calibration of two independent examiners (MH, AG) who marked the bone level points. Meanwhile, another calibrated examiner (VA) calculated the possible bone gain by taking linear measurements from the most mesial and distal part of the implant platform point to the crestal bone in each periapical radiograph, taking into account the height and diameter of each implant.

**Figure 3:**
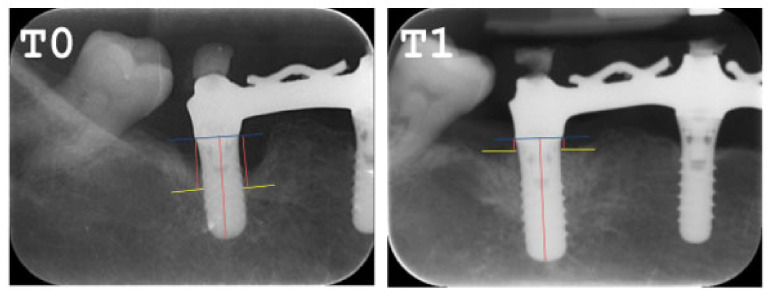
Periapical radiograph pre and post. T0: Periapical Rx pre regenerative surgery in peri-implant defects. T1: Periapical Rx pre regenerative surgery in peri-implant defects.

Radiographic bone gain was calculated in the T1 control radiograph, compared to the peri-implant bone defects of the T0 phase, as illustrated in the following image (Figure 3).

### 2.6. Non-Surgical Therapy Phase

After the diagnosis of peri-implantitis, oral hygiene education was provided to patients. All those who participated in the study received non-surgical treatment for at least 8 weeks prior to regenerative surgery. The treatment consisted of scaling and root planing, with ultrasound (Kavo Sonicflex^®^), mini curettes (Hu-Friedy), and irrigation of the peri-implant pockets with 0.12% chlorhexidine (Perioaid, Dentaid, Barcelona, Spain).

### 2.7. Surgical Reconstructive Phase

For the surgical phase, antibiotic prophylaxis was administered to the patient, amoxicillin 875/125 mg (Normon^®^, Madrid, Spain) + Metronidazole 250 mg (Normon^®^, Madrid, Spain), using two tablets 1 h before the intervention. A surgery access was performed with a full thickness flap to perform debridement of the granulation tissue using mini-five curettes (Hu-Friedy, Chicago, IL, USA). Surface detoxification was performed with 3–5% hydrogen peroxide for 2 minutes and irrigation with 0.12% chlorhexidine.

**Figure 4:**
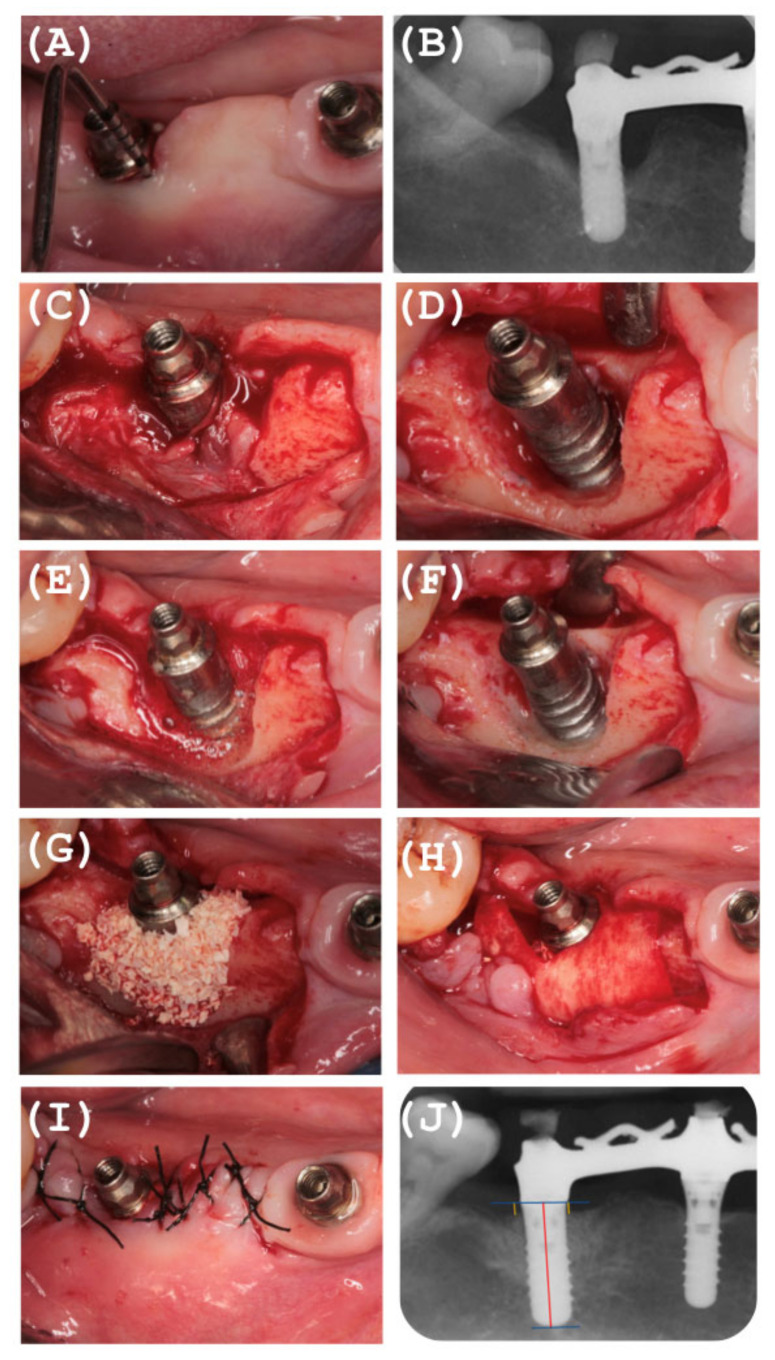
Illustrative case of the Surgical reconstructive approach. (**A**,**B**): Periimplantitis Diagnosed including Clinical and Radiological variables. (**C**): Surgery Access. (**D**): Mechanical Debridement. (**E**,**F**): Detoxification on implant Surface. (**G**): Deproteinized Bovine Bone Mineral. (**H**): Membrane Stability. (**I**): Primary Wound Healing. (**J**): Radiographic Hard Tissue Gain after 24 months.

The surgical approach was adapted to the clinical scenario, and the resective therapy was performed for supracrestal defects (15.6%), which were treated by guided bone regeneration and implantoplasty polishing the implant threads, with the aim of reducing the adherence capacity of the dental biofilm, while the peri-implant infrabony defects (84.4%) were two or three wall defects with loss of the vestibular table, which were filled with xenograft (Bio-Oss^®^, Geistlich Pharma, Wolhusen, Switzerland). A reabsorbable collagen membrane (Jason^®^, Botis, Berlin, Germany) was placed on the graft and adapted to the morphology of the defect, and where possible, it was stabilized with tacks (Klockner^®^, Soadco, Andorra). It was sutured with non-absorbable multifilament 5/0 (Supramid^®^, Braun, Kronberg, Germany) in cases where the prosthesis was removed and with resorbable polyglycolic acid 5/0 (Serapid^®^, Osteogens, Madrid, Spain) in cases where the prosthesis was not removed, ensuring the closure of the wound (Figure 4).

### 2.8. Postoperative Care

Patients were instructed to rinse the surgical area with 0.12% chlorhexidine twice a day for 2 weeks, in addition to amoxicillin and systemic metronidazole (3 tablets a day for 1 week). It was recommended that anti-inflammatory drug ibuprofen^®^ 600 mg be taken, 1 tablet every 8 h for 3 days. After 14 days, the sutures were removed; in cases where the prosthesis was removed, they were replaced 8–10 weeks after the regenerative surgery.

### 2.9. Recall Program

Patients were monitored after regenerative treatment on a maintenance program every 4 months. The radiographic bone filling was assessed a minimum of 24 months after the regenerative therapy. In cases where tissue hygienization was not possible due to the design of the prosthesis, a modification of the prosthesis was carried out until correct hygienic access was achieved.

### 2.10. Statistical Analysis

A descriptive analysis of all variables was carried out to present the data at the level of the 32 implants registered in the study in T0 and T1 as mean values (percentile values from 5% to 95%).

The following analyses were performed:

A complete descriptive analysis was made detailing all variables.
The Chi square test was carried out. In order to determine the groups that made the difference, Haberman’s corrected standardized waste was used, which allowed us to obtain the significance of the cells independently. This significance implies that the percentage of the cell is different, statistically, from that corresponding to the total of the sample.

Previously, a normality test had been applied, using, therefore, the ANOVA (variables of normal distribution).

## 3. Results

Initially, 30 patients were chosen to participate in the study, of which 30 were treated, but two of them did not attend the control after the surgery; therefore, these two patients were excluded from the study.

**Figure 5:**
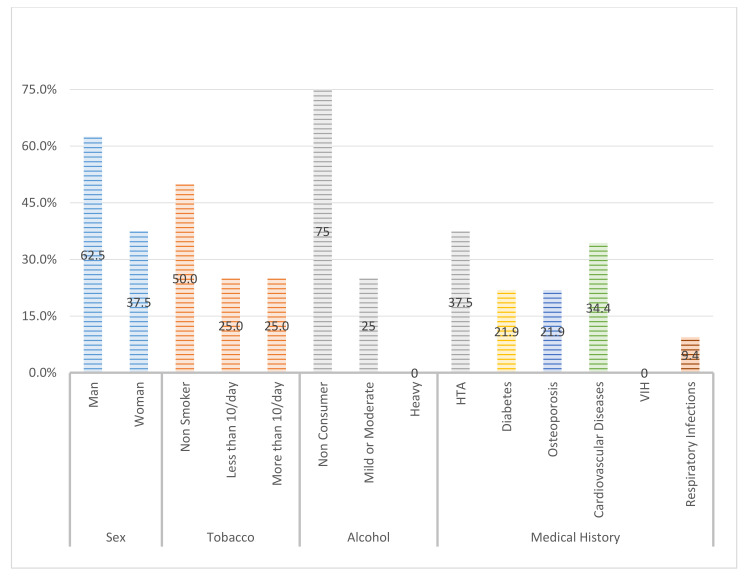
General variables registered in the patients.

A total of 28 patients (n: 32 implants) (62.5% men and 37.5% women) participated in the study. The average follow-up time for patients was 2.72 years (±1.44). The general variables registered in the patients can be seen in Figure 5, and other variables such as alcohol or tobacco habits are presented in Table 1.

Of the implants registered in the study, 62.5% were located in the maxilla and 37.5% were located in the mandible. Regarding the commercial brand of the implants, 68.75% were Straumann^®^ implants (Basel, Switzerland), while the remaining 31.25% were from other brands such as Klockner^®^ (Andorra, Principate of Andorra), Zimmer^®^ (Warsaw, Indiana, USA), and Nobel Biocare^®^ (Richmond Hill, Canada, USA). In terms of the type of implant registered, 59.4% had a polished neck design (Straumman Tissue Level^®^, Basel, Switzerland; Klockner Essential^®^, Andorra, Principate of Andorra), 25% were bone-level implants, and 5% were external connection implants. Regarding the diameter and length of the implants assessed, 81.3% had a diameter of 4 mm or more, while the other 18.8% corresponded to a diameter of less than 4 mm, 96.9% were implants of a length greater than or equal to 8 mm, and the remaining 3.1% were smaller than 8 mm. The surface of 100% of the included implants was moderately rough (1 to 2 µm).

Regarding the loading protocols for prosthetic rehabilitation, it was carried out conventionally (>2 months) in 65.6% of the cases and immediately (0–1 week) or early (1 week to 2 months), in 34.5%, according to the criteria of Espósito et al. [25]. The type of restoration of the implants in the study was mostly screw-fixed partial prosthesis (46.9%), followed by screw-fixed complete prosthesis (hybrid and metal–porcelain) in 28.1%, screwed individual crowns in 18.8%, and finally cemented individual crowns in 6.3%. Regarding the passive fit, 87.5% of the patients had a good fit, and in 68.8%, the prostheses were not overcontoured, in 71.9%, the antagonistic arches corresponded to natural teeth, 15.6% fixed prostheses on implants, and 12.5% fixed prostheses onto teeth. The differences of these qualitative variables between removal or not of the prosthesis after regenerative surgery in peri-implant defects are statistically insignificant (Table 2).

All of the implants in the study had peri-implantitis, presenting bleeding and suppuration after probing at clinical examination, and none of the implants had mobility in clinical phases T0 and T1.

There were no postoperative complications, such as membrane and graft exposure, or cases of early infection. All this led to no additional prescription of analgesics and antibiotics beyond the initial instruction recommended.

Clinical and Radiographic Changes.

**Figure 6:**
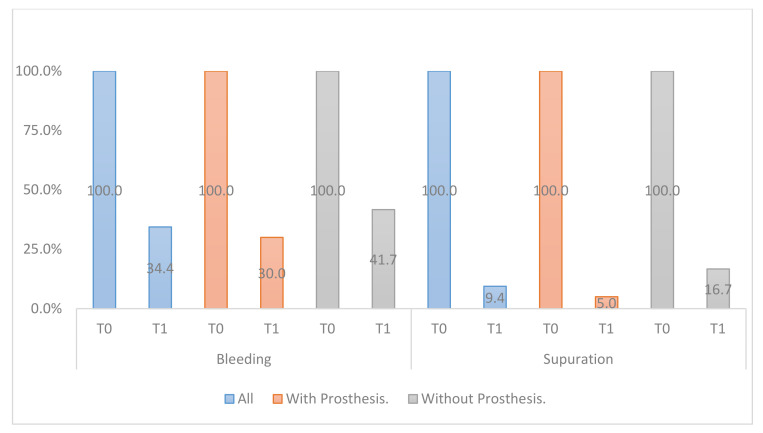
Percentage of bleeding and suppuration in T0, T1, differentiating between removing the prosthesis or not after regenerative surgery in peri-implant bone.

**Figure 7:**
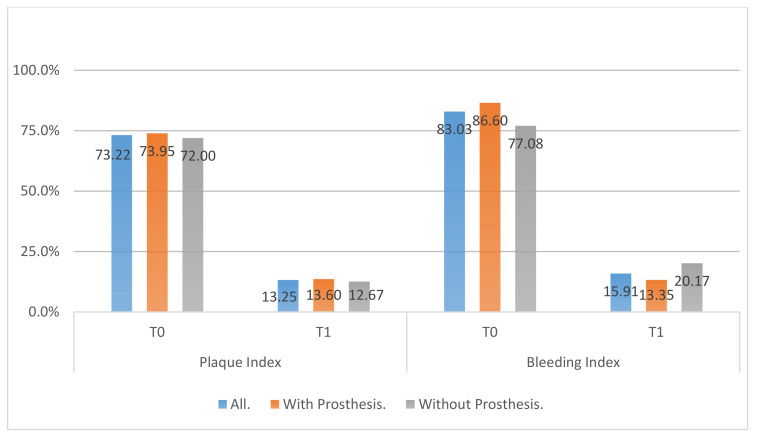
Plaque and bleeding index in T0, T1, differentiating between removing the prosthesis or not after regenerative surgery in peri-implant bone.

**Figure 8:**
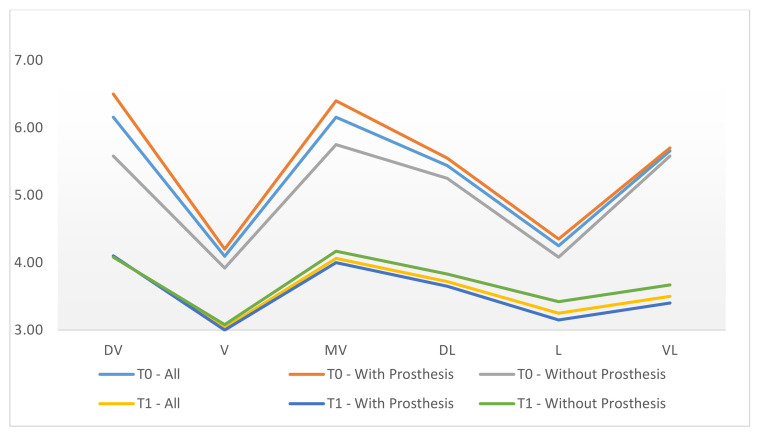
Differentiation of probing depth (PD) in phases T0, T1, comparing the prosthesis removal group after regenerative surgery in peri-implant bone with the non-removal group.

**Figure 9:**
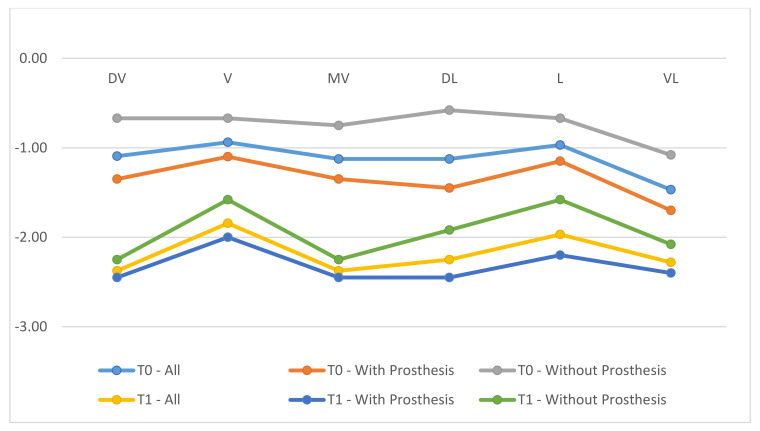
Differentiation of Clinical Attachment Level (CAL) in phases T0, T1, comparing the prosthesis removal group after regenerative surgery in peri-implant bone with the non-removal group.

- All clinical variables changed substantially from T0 to T1.
- PD, SUP, mPI, and mBI decreased from T0 to T1 (Figure 6, Figure 7, Figure 8 and Figure 9).
- REC increased from T0 to T1.

**Figure 10:**
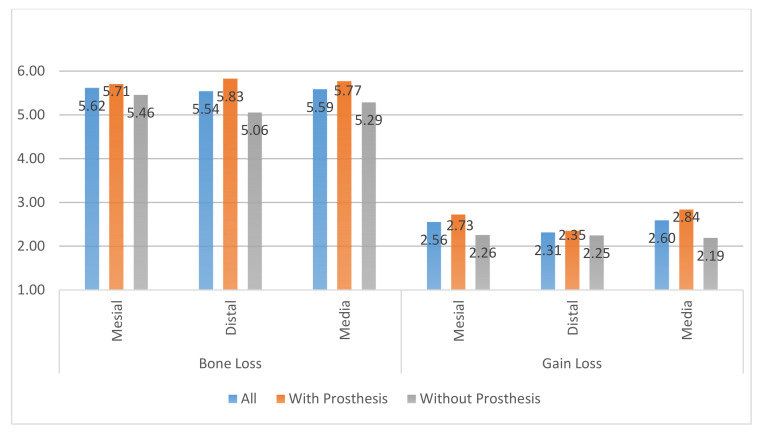
Comparison of radiographic bone loss (MBL) and gain values in cases where the prosthesis was maintained with cases where it was removed after regenerative surgery in peri-implant bone defects.

Regarding the removal of the prosthesis or not after regenerative surgery of the peri-implantary defects, it was kept in 62.5% of the cases, while in the remaining 37.5%, it was removed during the healing time. Radiographic parameters varied from T0 to T1 (Figure 10).

▪ The MBL decreased from T0 to T1.
▪ A radiographic bone filling was obtained in most cases.

### Satisfaction Questionnaire

**Figure 11:**
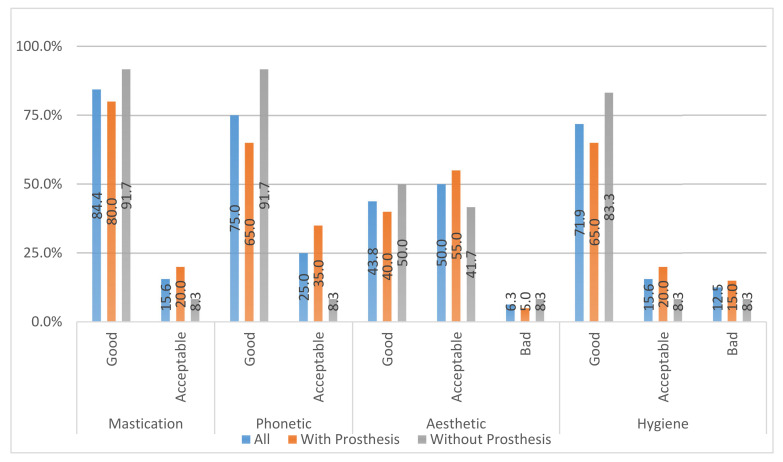
Questionnaire satisfaction of patients in both groups after regenerative surgery in peri-implant bone.

Variables such as mastication, phonetics, aesthetics, and cleaning (Figure 11) of implant prosthetic restorations were evaluated. In general, all patients were satisfied after receiving the treatment; however, they felt that there was an aesthetic implication of this therapy: because it is not advisable to expose the rough surface of the implants to the oral cavity, the supracrestal component has to be approached by implantoplasty, taking into account that a polished surface is more compatible with an inflammation-free zone due to the lower accumulation of biofilms. Therefore, it must be considered that this approach may have aesthetic implications in patients, which must be communicated to patients with high aesthetic demands.

## 4. Discussion

The main objective of this work is to determine, in the results of the treatment of peri-implant defects, whether or not removing the prosthesis has an influence when we carry out regenerative therapy in peri-implantitis. The objective of peri-implantitis treatment is to resolve the inflammation of the soft tissues and preserve the peri-implantary bone. The treatment of peri-implant disease is still a controversial issue. There is no “gold standard”, and several techniques have been recommended to treat peri-implantitis, from non-surgical procedures to complex resective and regenerative surgical procedures, which aim at preventing inflammation and encouraging the regeneration of hard and soft tissues surrounding the implant [26]. Therefore, it is essential to address appropriate treatment therapies for peri-implantitis, although the surgical technique and its result may vary according to the morphology of the defect involved.

The results of this series of cases provide potential benefits of regenerative therapy by guided bone regeneration to treat two or three-wall infrabony defects and suprabony defects.

Parameters such as mBI, mPI, and PD were higher in unresolved cases, but due to the small sample size, these do not have statistical significance. A greater number of randomized controlled clinical trials are needed to determine if the invasive surgical procedure described here provides superior results to less invasive procedures with respect to implant survival.

The proposed protocol presents similarities in reference to other studies published in the literature [10,19,27,28,29]. Positive results were obtained in terms of probing depth, reduction of bleeding, and radiographic bone filling of hard tissue, despite the fact that different post-operative therapies were applied, with or without the removal of the prosthesis during the healing phase.

The concept of submerged healing by removing the prosthetic restoration has been an alternative treatment since 1965 [20]. This therapeutic option is based on the hypothesis that aseptic healing following the paradigm of guided bone regeneration is crucial to stabilize a blood clot, thus improving the stability of the graft and maximizing the regenerative potential of the intraosseous compartment [30,31]. Khoury and Buchmann [32] obtained as results with this therapeutic option an average bone filling of 1.7–2.5 mm, using autologous bone grafts in 41 peri-implant defects treated in 25 patients. Other authors such as Schwarz et al. [29], in an animal study (30 implants in five beagles), concluded that submerged healing improved the outcome of the treatment, thus improving the chances of promoting reosseointegration compared to non-submerged healing.

Subsequently, Roos-Jansaker et al. [2,19] evaluated the result obtained after a regenerative treatment in peri-implant defects. After the removal of the prosthetic restoration between 3 and 6 months, they obtained an average reduction of the probing depth of 4.2 mm and a mean filling of the infra-bone defects of 2.3 mm.

Wiltfang et al. [33] carried out regenerative treatments in peri-implant defects, combining xenogeneic and autologous bone, without removing the prosthetic restoration. The results of this study were better compared to other studies in which only autologous bone was used [30]. They concluded that for bone defects of more than 4 mm in case of peri-implantitis, a single surgical intervention provided a reliable method to reduce bone defects, with non-submerged healing of peri-implant defects.

Recent meta-analyses, such as that of Lollobrigida et al. [34], revealed that there are only two studies [21,35] that compare the effect of healing in peri-implant defects, removing prosthetic restorations or not after regenerative surgery in peri-implantitis, showing better clinical results when submerged healing and barrier membranes were performed, and furthermore indicating that rough surfaces can improve reosseointegration when compared to smooth surfaces. This confirms what is described by Schwarz et al. [29], who found that submerged healing is preferable for several reasons, since there is complete isolation in the regenerated area, better soft tissue healing, and a decrease in the risk of postoperative infection.

However, due to the limited number of randomized clinical trials, currently, there is a lack of scientific evidence in the literature on the superiority of removing the prosthesis compared to not removing it during the healing process after regenerative treatment in peri-implantitis. No conclusions could be drawn due to the high heterogeneity between the current studies. Canullo et al. [21] deduced in a prospective study that they obtained better results when the prosthetic restoration was removed. Daugela et al. [35] reported no statistically significant differences between removing the prosthesis or not during the healing process. However, Wiltfang et al. [33] determined that they obtained better results after a peri-implant regenerative surgery when they maintained the prosthesis.

Including patients in a maintenance program is one of the main factors for long-term success in this type of treatment [36]. Furthermore, techniques for increasing the keratinized mucosa around implants have been shown to improve results after regenerative therapy in peri-implant defects and to improve bleeding and bacterial plaque rates, as reported by Khoury et al. [37].

Since there are no clear indications regarding the recovery intervals of the patients or the applied protocols (ultrasonic vs. manual debridement), no definitive conclusions can be obtained. All the patients included in the study were included in a maintenance program to ensure optimum control of the biofilms; the patients who did not have the best results after bone regeneration were those with the highest bacterial plaque index in T0 and T1.

To summarize the results obtained in this study, a comparison of two groups, one group of cases removing the prosthesis and another control group not removing it, obtained results without statistical significance during a minimum follow-up period of two years after the regenerative surgery of the peri-implantary bone defects.

## 5. Conclusions

Based on the results obtained, this technique suggests that the regenerative method, by means of xenograft, resorbable membrane, and detoxifying the implant surface with hydrogen peroxide, is a reliable procedure, with similar bone gain results in both groups for achieving medium-term results.

Based on this study, there are no statistically significant differences in response to treatment whether or not the prosthesis is removed; therefore, we can conclude that the success of the treatment does not depend on this factor once the bone regenerative surgery of the peri-implant defects has been performed. This surgical approach triggers positive effects on the different clinical parameters, such as reduction in bleeding and bacterial plaque, probing depth, clinical attachment gain, and radiographic bone filling during the follow-up period.

## Figures and Tables

**Table 1:** Influence of smoking and alcohol habits on results after removal or not of the prosthesis in regenerative treatments in peri-implant bone defects.

Variable	Rank	Yes		No		Sign.
		Frec.	Porc.	Frec.	Porc.	
Tobacco	Nonsmoker	7	43.8	9	56.3	0
	Less than 10 cig/day	6	75	2	25	0
	More than 10 cig/day	7	87.5	1	12.5	0
Alcohol	Non-Consumer	14	58.3	10	41.7	0
	Mild or Moderate Consumer	6	75	2	25	0
	Heavy Consumer	0	---	0	---	0

**Table 2:** Influence of removal or not of the prosthesis after peri-implant bone regenerative therapy, according to qualitative variables related to the implant. ^(S)^ Although significance is detected in the table, as a whole, the analysis of the cells does not reflect any relevant concrete differences.

Variable	Rank	Yes		No		Sign.
		Frec.	Porc.	Frec.	Porc.	
Type of Prosthetic Restoration	Cemented Retained	2	100	0	0	<0.05 ^(s)^
	Screw Retained	18	100	12	100	0
Passive Fit	Yes	18	64.3	10	35.7	0
	No	2	50	2	50	0
Prosthetic Contour	Yes	6	60	4	40	0
	No	14	63.6	8	36.4	0
Antagonistic Arcade	Natural Teeth	13	56.5	10	43.5	0
	Fixed Dental Prostheses	3	75	1	25	0
	Fixed Implant Supported	4	80	1	20	0

## Data Availability

The data presented in this study are available on request from the corresponding author. The data are not publicly available due to privacy.
